# Analysis of factors contributing to occupational health inequality in Korea: a cross-sectional study using nationally representative survey data

**DOI:** 10.1186/s13690-021-00638-9

**Published:** 2021-06-23

**Authors:** Eunjeong Noh, Young-Ho Khang

**Affiliations:** 1 Institute of Health Policy and Management, Medical Research Center, Seoul National University, Seoul, Republic of Korea; 2 Department of Health Policy and Management, College of Medicine, Seoul National University, Seoul, Republic of Korea

**Keywords:** Health inequalities, Mortality, Occupations, Psychology, Republic of Korea, Risk factors, Socioeconomic factors

## Abstract

**Background:**

Despite the consensus that higher occupational classes tend to have better health and lower mortality rates, one study has reported reversed occupational gradients in mortality rates among Korean men after the economic crisis in the late 2000s. To examine these patterns of health inequality in more detail, we investigated the tendency of occupational gradients in socioeconomic position and multiple pathway indicators known to affect mortality in Korea.

**Methods:**

We used data from 4176 men aged 35–64 in Korea derived from the 2007–2009 and 2013–2015 Korean National Health and Nutrition Examination Surveys. We compared the age-standardized prevalence and age-adjusted mean values of each contributing factor to health inequality among occupational groups, which are divided into upper non-manual workers, lower non-manual workers, manual workers, and others. Contributing factors included childhood and adulthood socioeconomic position indicators, biological risk factors, health behaviors, psychosocial factors, and work environment.

**Results:**

Upper non-manual workers had prominently higher levels of education, income, parental education, and economic activity than lower non-manual and manual workers. The rates of smoking and high-risk alcohol consumption were lower, and the rate of weight control activities was higher, in the non-manual classes. Further, the rates of depression and suicidal ideation were lower, and perceptions of the work environment were more favorable, among non-manual workers than among their manual counterparts.

**Conclusions:**

We detected occupational inequality in a wide range of socioeconomic positions and pathway indicators in Korea with consistently favorable patterns for upper non-manual workers. These occupational gradients do not support the previously reported reversed pattern of higher mortality rates in non-manual groups versus in the manual job class in Korea.

**Supplementary Information:**

The online version contains supplementary material available at 10.1186/s13690-021-00638-9.

## Background

Several international studies have investigated health gradients according to occupational status, with generally concordant findings of better health and lower mortality among people with a higher occupational status. In other words, people with non-manual jobs (e.g., professional and office jobs) have lower mortality and morbidity as well as better health behaviors than those who work in jobs with high levels of manual labor [1–7]. However, Tanaka et al. argued that occupational gradient patterns in mortality in Korea have diverged from European countries since the economic crisis of the 2000s [8]. They claimed that since this time, the all-cause mortality rate for people with upper non-manual jobs has increased dramatically, exceeding that of people with lower non-manual and manual jobs. They proposed that the economic crisis might have affected the rising prevalence of cancer and suicide among non-manual workers, thereby contributing to the reversed patterns of health inequalities [8–10]. Mackenbach also stated in his recent book that “the main exception is Japan and South Korea where occupational class inequalities in mortality do not follow the usual pattern” (p. 47) [10]. The deterioration of factors contributing to health in response to the economic crisis might explain the anomalous occupational gradients in Korea. However, Khang pointed out that these purportedly reversed patterns are likely to be biased due to the use of unlinked data with population denominators from census data and mortality numerators from death certificates [11]. Previous Korean studies have also shown that using unlinked data may result in increased mortality estimates among non-manual workers and reduced mortality estimates among manual workers [12]. Moreover, the category of an “inactive or class-unknown” group, which accounted for 44–51% of total deaths in the most recent decade in Korea, may be related to the reversed pattern that Tanaka et al. and Tanaka reported [8, 9].

Although this finding of reversed health inequality patterns in Korea poses an important challenge to the universality of health inequality by occupational class, little research has clearly identified the unusual patterns of occupational inequality in mortality in Korea [10]. To shed further light on this controversy, we investigated the tendencies of occupational inequality in socioeconomic environments and various pathway factors known to affect mortality. If the contributing factors to occupational health inequalities reveal better conditions among the upper non-manual class than in lower non-manual and manual workers, these findings could suggest that the reversed health inequality in Korea is likely due to other issues (e.g., use of unlinked data) rather than to changes in the status of contributing factors influenced by the economic crisis [8].

## Methods

### Data sources and population

We used the Korea National Health and Nutrition Examination Survey (KNHANES) IV, which is a nationally representative cross-sectional survey conducted by the Korea Centers for Disease Control and Prevention (KCDC). The survey includes information on socioeconomic status, health-related behaviors, quality of life, healthcare utilization, anthropometric measures, and biochemical and clinical profiles collected through three component surveys: a health interview, a health examination, and a nutrition survey [13, 14]. In particular, the 2007–2009 KNHANES IV contains information on participants’ work environment. Therefore, these data constitute an appropriate source for investigating occupational gradients in various pathway variables known to influence health status and to contribute to mortality in Korea. We also employed the more recent 2013–2015 KNHANES data, which lacked work environment variables, for an additional analysis of occupational inequalities in contributing factors to mortality in Korea. The Institutional Review Board (IRB) of the KCDC approved these nationwide studies. Our study was exempted from IRB approval by the Seoul National University Hospital IRB (IRB number: E-2005-091-1123).

The KNHANES IV was conducted among noninstitutionalized Korean citizens residing in Korea from 2007 to 2009. Of the 31,705 targeted individuals, 24,871 participated in the survey (participation rate = 78.4%). Because Tanaka et al.’s 2019 study considered the association between occupational class and mortality among men aged 35–64, we focused on the same population—men aged 35 to 64 years in Korea—to enable equivalent comparisons [8]. Ultimately, we included 4176 men in the analyses, excluding all female participants as well as men outside the age range of 35–64. We extracted demographic information (age) and variables of interest (e.g., occupational class, socioeconomic position, and other pathway variables) from the dataset (see Additional file 1: Table S1).

### Occupational class and contributing factors to health inequality

We collected the data on occupational class and contributing variables to health from the 2007–2009 KNHANES. We categorized occupational class into four groups using the same definition as in Tanaka et al.’s study: upper non-manual workers, lower non-manual workers, manual workers, and others (e.g., agricultural, forestry and fishery workers, and unemployed people) [8]. We also classified socioeconomic and pathway variables into six categories—childhood socioeconomic position (SEP), adulthood SEP, biological health risk factors, health behaviors, psychosocial factors, and work environment—to explore the patterns of factors contributing to occupational health inequalities. For childhood SEP, we looked at parents’ education level, the absence of parents, and adult height, all of which numerous studies have confirmed as valid indicators for early life exposure measures [10, 15–21]. We used monthly household income and education level for adulthood SEP and body mass index (BMI), systolic blood pressure, total cholesterol, and glucose levels as the main biological risk factors. We considered four measures to investigate health behaviors: smoking, alcohol consumption, physical activity, and exercise for weight management. Psychosocial factors included feelings of depression for more than 2 weeks, stress awareness, marital status, and suicidal ideation. Finally, we analyzed responses to eight survey items asking about respondents’ work environment.

### Statistical analysis

The KNHANES data were collected through a complex multistage probability sample design [14] to improve the representativeness of the samples and accuracy of the estimations. In the complex multistage sampling design, the estimated association may be biased by the oversampled subgroups of the population. To resolve such issue, it is recommended to use the complex sample analysis method to apply weight to individual factors [22]. Thus, we analyzed the data using the complex sample analysis method that took into account the three elements of strata, clustering, and sample weight.

We compared the prevalence (for categorical variables) and mean values (for continuous variables) of each contributing variable among occupational groups to investigate occupational gradients. Because age is an important confounder in the association between SEP and health indicators, we present the resultant statistics as age-standardized rates calculated with the direct standardized method using data from the 2010 population census as the reference population. We also provide the least squared means by using the *proc surveyreg* procedure in SAS to determine the age-adjusted means of the variables. We then estimated the 95% confidence intervals of the adjusted prevalence and mean values by occupational class. We conducted the data processing and statistical analysis using SAS version 9.4 (SAS Institute, Cary, NC, USA).

## Results

We examined whether gaps in SEP and pathway variables existed depending on occupational status (Table 1 and Table 2). In terms of childhood SEP, 43.2% of upper non-manual workers’ fathers had a primary education or below, compared to 52.2% of lower non-manual workers and 59.1% of manual workers. In total, 62.5% of upper non-manual workers’ mothers had a primary education or below, compared to 68% of lower non-manual workers and 72.6% of manual workers. The rate of parents’ absence in childhood was highest in manual workers (10.3% for upper non-manual, 11.6% for lower non-manual, and 13.9% for manual workers). Average adult height was 169.8 cm in upper non-manual, 169.7 cm in lower non-manual, and 168.1 cm in manual workers (Table 2).

**Table 1:** Age-standardized percentages of childhood and adulthood socioeconomic position (SEP) indicators and pathway variables by occupational class for men aged 35–64: The 2007–2009 Korea National Health and Nutrition Survey (*n* = 4176; weighted *n* = 10,528,165)

		Upper Non-manual		Lower Non-manual		Manual		Others	
		%^**c**^ (n)^**d**^	95% CI	% (n)	95% CI	% (n)	95% CI	% (n)	95% CI
**Childhood SEP**	**Parents’ education level**								
	Father’s education (elementary school or less)	43.2 (304)	38.8–47.7	52.2 (494)	48.6–55.7	59.1 (822)	56.3–62.0	56.2 (628)	51.9–60.5
	Mother’s education (elementary school or less)	62.5 (456)	58.6–66.3	68.0 (662)	64.6–71.4	72.6 (1023)	69.9–75.3	70.8 (739)	66.5–75.0
	**Parents’ economic activity**								
	Absence of parents	10.3 (65)	7.5–13.0	11.6 (105)	9.2–14.1	13.9 (190)	11.9–15.9	13.3 (157)	10.5–16.1
**Adulthood SEP**	**Education level**								
	Elementary school or less	2.8 (21)	1.4–4.3	4.3 (45)	2.9–5.8	18.5 (270)	16.2–20.7	27.3 (289)	24.0–30.6
	Middle school	1.4 (9)	0.4–2.3	8.8 (85)	6.8–10.7	21.0 (297)	18.5–23.5	22.4 (229)	19.3–25.5
	High school	23.8 (169)	20.0–27.6	38.3 (383)	34.8–41.8	48.4 (658)	45.3–51.5	33.3 (290)	29.6–37.1
	College or above	72.0 (554)	68.0–76.0	48.6 (491)	44.9–52.4	12.2 (166)	10.3–14.0	17.0 (145)	13.6–20.3
**Biological health risk factors**	Body mass index (BMI) (≥30 kg/m^2^)	4.1 (33)	2.7–5.5	2.9 (31)	1.8–4.1	2.7 (34)	1.7–3.7	4.2 (28)	2.0–6.5
	BMI (≤18 kg/m^2^)	2.1 (17)	1.1–3.2	2.3 (27)	1.3–3.2	3.4 (50)	2.3–4.4	5.8 (53)	3.7–7.9
	Blood pressure (≥140 mmHg)	5.0 (36)	3.2–6.8	7.1 (58)	5.0–9.1	10.3 (140)	8.5–12.1	11.9 (131)	8.9–15.0
	Serum total cholesterol (≥240 mg/dL)	7.6 (64)	5.6–9.7	6.9 (76)	5.3–8.5	8.7 (117)	6.9–10.6	8.0 (79)	5.8–10.3
	Serum glucose level (≥126 mg/dL)	8.0 (54)	5.8–10.2	8.1 (72)	5.9–10.3	9.1 (125)	7.3–10.8	8.7 (91)	6.2–11.1
**Health behaviors**	Current smoking	41.3 (319)	37.2–45.4	40.8 (444)	37.4–44.2	51.0 (686)	48.2–53.8	49.3 (411)	44.9–53.8
	High-risk alcohol consumption^**a**^	9.1 (69)	6.8–11.4	11.4 (111)	9.0–13.8	14.3 (190)	12.2–16.3	12.9 (120)	10.2–15.5
	Moderate level of physical activity	9.3 (75)	7.0–11.6	11.4 (121)	9.1–13.7	17.4 (263)	15.3–19.6	13.3 (160)	10.4–16.2
	Exercise for weight control	52.2 (392)	48.3–56.2	45.8 (466)	42.2–49.3	34.5 (484)	31.6–37.3	35.9 (324)	31.5–40.4
**Psychosocial factors**	Feelings of depression (more than 2 weeks)	9.3 (74)	7.1–11.6	9.5 (92)	7.4–11.7	9.8 (143)	8.2–11.3	16.2 (140)	12.8–19.6
	Stress awareness	31.8 (255)	28.2–35.4	30.6 (343)	27.5–33.6	23.3 (332)	20.8–25.9	23.4 (195)	19.4–27.5
	Marital status (yes)	97.6 (732)	96.7–98.6	97.7 (974)	96.8–98.6	94.9 (1318)	93.6–96.2	81.8 (876)	77.9–85.6
	Suicidal ideation	7.3 (60)	5.4–9.3	10.1 (92)	7.6–12.5	10.8 (153)	9.0–12.5	18.7 (159)	15.0–22.4
**Work environment** ^**b**^	Cleanliness and comfort	88.4 (666)	85.6–91.2	85.8 (866)	83.2–88.4	54.9 (784)	52.1–57.7	34.6 (361)	30.2–38.9
	Dangerous	18.5 (140)	15.3–21.8	18.3 (199)	15.6–21.1	62.7 (867)	59.7–65.7	23.4 (238)	19.6–27.3
	Time pressure	36.9 (296)	33.2–40.5	35.9 (393)	32.6–39.2	36.5 (505)	33.8–39.1	25.1 (257)	21.0–29.1
	Authority	95.2 (718)	93.5–96.9	89.1 (897)	86.7–91.5	68.4 (960)	65.6–71.1	46.8 (472)	41.3–52.2
	Respected and trusted	92.4 (697)	90.1–94.7	91.8 (924)	89.8–93.8	85.7 (1188)	83.7–87.7	47.1 (489)	41.7–52.6
	Long hours in an uncomfortable position	9.4 (80)	7.4–11.5	16.4 (156)	13.7–19.1	36.7 (522)	33.9–39.5	22.7 (251)	18.2–27.2
	Carrying heavy objects	7.8 (58)	5.6–10.0	22.7 (207)	19.5–25.8	39.7 (560)	36.5–42.8	25.5 (290)	21.5–29.5
	Hiding emotions	34.2 (272)	30.4–38.0	35.8 (379)	32.4–39.2	37.9 (529)	35.1–40.8	16.7 (144)	13.2–20.2

^a^Drinking more than seven drinks per day on average almost every day in the past year

^b^The proportion of those who responded “agree” or higher for each question about their working environment (strongly disagree = 1, disagree = 2, agree = 3, strongly agree = 4)

^c^Age-standardized prevalence percentage calculated based on the weighted frequencies

^d^Sub-sample population size without applying weight

**Table 2:** Age-adjusted means^a^ of childhood and adulthood socioeconomic position (SEP) indicators and pathway variables by occupational class for men aged 35–64: The 2007–2009 Korea National Health and Nutrition Survey (*n* = 4176; weighted *n* = 10,528,165)

		Upper Non-manual		Lower Non-manual		Manual		Others	
		Mean	95% CI	Mean	95% CI	Mean	95% CI	Mean	95% CI
**Childhood SEP**	Height (cm)	169.8	169.4–170.3	169.7	169.4–170.1	168.1	167.7–168.4	168.4	167.9–168.9
**Adulthood SEP**	Monthly household income (10,000 won) ^**b**^	424.9	404.1–445.8	354.1	338.0–370.2	272.8	261.3–284.2	210.6	194.5–226.7
**Biological health risk factors**	Body mass index (kg/m^2^)	24.7	24.5–24.9	24.5	24.3–24.8	24.1	23.9–24.3	24.0	23.8–24.2
	Blood pressure (mmHg)	117.5	116.1–119.0	118.9	117.8–120.0	120.6	119.5–121.7	120.6	119.2–121.9
	Serum total cholesterol (mg/dL)	192.1	189.5–194.7	189.9	187.4–192.3	191.8	189.5–194.1	191.0	188.2–193.8
	Serum glucose level (mg/dL)	102.4	100.6–104.2	102.5	100.9–104.0	102.7	101.0–104.4	102.2	100.1–104.3
**Health behaviors**	Smoking quantity per day (cigarettes)	17.8	16.6–19.1	18.5	17.4–19.5	19.4	18.7–20.2	18.6	17.5–19.6
**Work environment** ^**c**^	Cleanliness and comfort	3.1	3.1–3.2	3.0	3.0–3.1	2.5	2.5–2.6	2.6	2.6–2.7
	Dangerous	1.7	1.7–1.8	1.8	1.8–1.9	2.6	2.6–2.7	2.3	2.2–2.4
	Time pressure	2.3	2.3–2.4	2.3	2.2–2.3	2.3	2.2–2.3	2.4	2.4–2.5
	Authority	3.2	3.2–3.3	3.0	3.0–3.1	2.7	2.7–2.8	3.0	3.0–3.1
	Respected and trusted	3.1	3.0–3.1	3.0	3.0–3.1	2.9	2.9–3.0	3.0	2.9–3.0
	Long hours in an uncomfortable position	1.8	1.8–1.9	1.9	1.9–2.0	2.3	2.3–2.4	2.4	2.3–2.5
	Carrying heavy objects	1.6	1.5–1.6	1.9	1.8–2.0	2.3	2.3–2.4	2.5	2.4–2.5
	Hiding emotions	2.2	2.2–2.3	2.3	2.2–2.3	2.3	2.3–2.3	2.1	2.1–2.2

^a^Age-adjusted (50 years old) least squared means

^b^Bottom-coding for less than 170,000 won per month and top-coding for more than 9 million won per month were applied

^c^Mean score of responses for each question about the working environment (strongly disagre = 1, disagree = 2, agree = 3, strongly agree = 4)

Regarding adulthood SEP, 2.8% of upper non-manual respondents had an elementary school or lower education, compared to 4.3% of lower non-manual and 18.5% of manual workers. In contrast, 72% of upper non-manual respondents had a college degree or higher, considerably exceeding the proportions among lower non-manual and manual worker respondents (48.6 and 12.2%, respectively) (Table 1). The average monthly household income was 4.25 million won for upper non-manual workers, 3.54 million won for lower non-manual workers, and 2.73 million won for manual workers (Table 2).

The proportions of individuals with a BMI exceeding 30 kg/m^2^ were 4.1, 2.9, and 2.7% of upper non-manual, lower non-manual, and manual workers, respectively. Meanwhile, those who had a BMI of less than 18 kg/m^2^ accounted for 2.1, 2.3, and 3.4% of upper non-manual, lower non-manual, and manual workers, respectively. Blood pressure readings of 140 mmHg and over were 5, 7.1, and 10.3% of upper non-manual, lower non-manual, and manual workers, respectively. Cholesterol levels exceeding 240 mg/dL were found in 7.6% of upper non-manual workers, 6.9% of lower non-manual workers, and 8.7% of manual workers. High glucose levels (≥126 mg/dL) were found in 8% of upper non-manual workers, 8.1% of lower non-manual workers, and 9.1% of manual workers. Except for BMI, high-risk status for these indicators tended to be more common in manual versus non-manual workers (Table 1).

The average smoking quantity per day (19.4 cigarettes) and smoking rate (51%) were both higher in manual workers than in non-manual workers (Table 1 and Table 2). High-risk alcohol consumption was also more common in manual workers (14.3%) than in upper (9.1%) or lower (11.4%) non-manual workers. Manual workers also tended to report a higher percentage of physical activity (17.4%) than upper (9.3%) or lower (11.4%) non-manual workers. However, exercise for weight control was more common in non-manual workers (upper: 52.2%, lower: 45.8%) than in manual workers (34.5%) (Table 1).

The percentage of those who experienced sustained feelings of depression was only slightly higher among manual workers (9.8%) than among upper (9.3%) and lower (9.5%) non-manual workers. Stress awareness rates were higher in non-manual workers (upper: 31.8%, lower: 30.6%) than in manual workers (23.3%). The marriage rate was higher among non-manual workers. In total, 97.6% of upper and 97.7% of lower non-manual workers were likely to receive social support through marriage, compared to 94.9% of manual workers. Suicidal ideation was more common in lower non-manual (10.1%) and manual (10.8%) workers than in upper non-manual workers (7.3%) (Table 1).

In terms of work environment, more non-manual workers agreed or strongly agreed that their workplaces were clean and comfortable (upper: 88.4%, lower: 85.8%) than manual workers (54.9%). Far more manual workers (62.7%) agreed or strongly agreed that they worked in a dangerous environment than non-manual workers. However, the proportion of respondents who agreed or strongly agreed they faced time pressure did not differ significantly among the three occupational groups (upper non-manual: 36.9%, lower non-manual: 35.9%, manual: 36.5%). Regarding having authority at work, more non-manual workers agreed or strongly agreed they had it (upper: 95.2%, lower: 89.1%) than manual workers (68.4%). Those who agreed or strongly agreed that they were respected and trusted were more likely to be non-manual workers (upper: 92.4%, lower: 91.8%) than manual workers (85.7%). More manual workers responded that they worked long hours in an uncomfortable position and carried heavy objects. Finally, more respondents who agreed or strongly agreed that they hid their emotions were manual workers (37.9%) than non-manual workers (upper: 34.2%, lower: 35.8%) (Table 1).

We found similar results in an additional analysis of the 2013–2015 KNHANES data containing the same variables on childhood and adulthood SEP indicators and pathway variables but that lacked work environment variables (see Additional file 1: Table S2 and Table S3).

## Discussion

We found a distinct occupational gap in childhood and adulthood SEP. Non-manual worker groups tended to have a better childhood and adulthood SEP than manual workers. The patterns of adulthood SEP by occupational status are not surprising. The results are in line with existing studies that report close correlations among high education levels, non-manual job choices, and higher income [6, 23, 24].

Except for BMI, we also found moderate occupational gaps in biological health risk factors. Systolic blood pressure, cholesterol, and glucose were higher in manual workers than in non-manual workers. We presume that these high levels of risk factors indicate greater exposure for manual workers, although the occupational gaps in these risk factors were smaller than those for childhood and adulthood SEP.

Non-manual workers reported healthier behaviors—namely, lower rates of smoking and high-risk drinking—than manual workers. Previous studies have also reported a lower age-standardized cancer mortality rate in upper non-manual workers (e.g., legislators, managers, and professionals) than manual workers [25]. In particular, higher incidence of and mortality from smoking- or alcohol-related cancers may be influenced by health behaviors linked with a lower SEP [25]. When these findings are taken together, the results of our analysis cannot support Tanaka et al.’s argument [8] that higher mortality among upper non-manual workers versus lower occupational classes may be affected by rising cancer-related mortality among upper non-manual workers. However, in terms of other health behavior measures, the proportion of manual workers who engaged in physical activities was higher. The 2007–2009 KNHANES defined physical activity as moderate-intensity physical activities performed at least 5 days a week for 30 min or more per day. Manual workers’ occupational physical activities may positively influence their health. Previous studies reported that non-sedentary physical activity may have a positive effect on workers’ physical health [26]. Further, workers who engaged in physically demanding occupations had a lower prevalence of obesity than those who did not [27]. An additional analysis of the 2013–2015 KNHANES data showed that physical activity for leisure was more common in higher occupational classes (30.7% in upper non-manual, 30.0% in lower non-manual, and 16.2% in manual workers) (Additional file 1: Table S2 and Table S3). Physical activity for weight control was also more common in non-manual versus manual workers.

Except for stress awareness, almost all the psychosocial factors showed disadvantageous conditions of manual workers (i.e., higher rates of depression and suicidal ideation and a lower marriage rate). SEP-related factors such as lower education, income, and employment status are well-known risk factors for suicide [25, 28–30]. We did not find distinct evidence of deterioration in SEP or psychosocial factors within the non-manual classes.

The proportion of workers who felt that their work environment was clean and pleasant, who had sufficient decision-making authority, and who felt respected and trusted was much higher among non-manual workers than manual workers. In contrast, higher proportions of manual workers carried out dangerous work, worked long hours in uncomfortable positions, carried heavy items, and hid their emotions. Thus, non-manual workers had more favorable perceptions of their work environment than manual workers, even after the economic crisis in Korea; it is unlikely that the incident has spurred the poor health at work and the rise in mortality of non-manual class. Prior studies have reported that low job control, measured by self-reported items on decision-making authority and skill discretion, significantly contributed to the employment gradient in the frequency of coronary heart disease [31]. Other research has shown that work stress, job strain, and effort–reward imbalance were related with the risk of cardiovascular death, with the association often confounded by SEP-related factors such as occupational class [6, 32, 33]. Based on our analysis and these previous studies, we cannot conclude that worse workplace conditions and increased psychosocial job stress caused by the economic crisis are linked to higher mortality among non-manual workers.

Although it raises important issues that are contrary to the conclusion suggested in Tanaka et al.’s study, our study has some limitations. We only used 3 years of data from the 2007–2009 KNHANES; thus, the results are limited in their explanation of the continuing effects of the economic crisis in Korea. However, our additional analyses of the 2013–2015 KNHANES data, excluding aspects of the work environment that were not collected in the surveys during those years, showed very similar tendencies of contributing factors by occupation class when compared to the results for 2007–2009 (see Additional file 1: Table S2 and Table S3). These results further corroborate our argument that the health status or mortality of non-manual workers may not have been more negatively affected by the economic crisis than that of manual workers in Korea. Furthermore, since we did not use the same predictors, outcome variables, and methodology as Tanaka et al., it is difficult for our results to challenge reversed health inequalities among occupational classes in a direct comparison with Tanaka et al.’s findings. While Tanaka et al. analyzed inequalities in mortality rates by occupational status, we observed inequality patterns in factors contributing to health by occupational class. Our work suggests that the health status of the manual or lower non-manual classes in Korea is still less likely to be better than that of the upper non-manual class. These findings allow us to infer that Tanaka et al.’s conclusions, that the mortality rate of the upper non-manual workers may be higher than that of the manual or lower non-manual classes, may be due to other problems (e.g., the use of unlinked data resulting in numerator-denominator bias), and not to the changes of psychosocial or environmental factors caused by the economic crisis. If mortality follow-up data are used to further investigate this issue in Korea, a more direct explanation could be developed for mortality patterns by occupational class, moving beyond an implicit interpretation based on the tendencies of contributing factors to health inequality.

## Conclusion

Overall, almost all the contributing factors showed better conditions in the non-manual occupational classes than in the manual class. Smoking, alcohol consumption, and weight control activities tended to be worse, and the rates of depression and suicidal ideation were higher, among manual workers. Work environment was more likely to be supportive of physical and psychological health for non-manual workers than for manual workers. Childhood and adulthood SEP also showed more pronounced gaps by occupational class than other factors. Those with non-manual jobs had a better current socioeconomic status and generally had a better childhood socioeconomic environment than those with manual jobs. SEP is not likely to work in a way that exacerbates the health conditions and mortality of non-manual workers. In conclusion, there is little evidence that SEP and pathway factors that change in response to influential events such as the economic crisis were worse among upper non-manual workers than among lower non-manual and manual workers. Instead, the numerator–denominator bias caused by using unlinked data is the most likely reason for the anomalous occupational gradients in mortality in Korea.

## Supplementary Information

**Additional file 1: Table S1.** Percentages and means of occupational class, childhood and adulthood socioeconomic position (SEP) indicators, and pathway variables for men aged 35–64: the 2007–2009 Korea National Health and Nutrition Survey (*n* = 4176). **Table S2.** Age-standardized percentages of childhood and adulthood socioeconomic position (SEP) indicators and pathway variables by occupational class for men aged 35–64: The 2013–2015 Korea National Health and Nutrition Survey (*n* = 4011). **Table S3.** Age-adjusted means^a^ of childhood and adulthood socioeconomic position (SEP) indicators and pathway variables by occupational class for men aged 35–64: the 2013–2015 Korea National Health and Nutrition Survey (*n* = 4011).

## Data Availability

Data sharing statement: The data used are publicly available from the Korea Centers for Disease Control and Prevention.

## Abbreviations

BMI: Body Mass Index
IRB: Institutional Review Board
KCDC: Korea Centers for Disease Control and Prevention
KHIDI: Korea Health Industry Development Institute
KNHANES: Korea National Health and Nutrition Examination Survey
SEP: Socioeconomic Position

## References

[1] Macintyre S (1997). The black report and beyond what are the issues?. Soc Sci Med.

[2] Marmot MG, McDowall ME (1986). Mortality decline and widening social inequalities. Lancet..

[3] Son M, Armstrong B, Choi JM, Yoon TY (2002). Relation of occupational class and education with mortality in Korea. J Epidemiol Community Health.

[4] Khang YH, Kim HR. Socioeconomic inequality in mortality using 12-year follow-up data from nationally representative surveys in South Korea. Int J Equity Health. 2016;15(1):1–11. 10.1186/s12939-016-0341-9. 10.1186/s12939-016-0341-9 PMC4802872 27001045

[5] Stringhini S, Sabia S, Shipley M, Brunner E, Nabi H, Kivimaki M (2010). Association of socioeconomic position with health behaviors and mortality. J Am Med Assoc.

[6] Khang YH, Lynch JW, Yang S, Harper S, Yun SC, Jung-Choi K, et al. The contribution of material, psychosocial, and behavioral factors in explaining educational and occupational mortality inequalities in a nationally representative sample of south Koreans: relative and absolute perspectives. Soc Sci Med. 2009;68(5):858–66. 10.1016/j.socscimed.2008.12.003. 10.1016/j.socscimed.2008.12.003 19121885

[7] Khang YH, Kim HR (2005). Relationship of education, occupation, and income with mortality in a representative longitudinal study of South Korea. Eur J Epidemiol.

[8] Tanaka H, Nusselder WJ, Bopp M, Brønnum-Hansen H, Kalediene R, Lee JS (2019). Mortality inequalities by occupational class among men in Japan, South Korea and eight European countries: a national register-based study, 1990-2015. J Epidemiol Community Health.

[9] Tanaka H. Re: mortality inequalities by occupational class among men in Japan, South Korea and eight European countries: a national register-based study, 1990–2015. J Epidemiol Community Health. 2019; Available from: https://jech.bmj.com/content/early/2019/06/07/jech-2018-211715.responses?versioned=true#the-surprising-result-of-manual-workers-in-korea-enjoying-lower-mortality-than-non-manual-workers-is-likely-due-to-numerator-denominator-bias. 10.1136/jech-2018-211715 PMC6678055 31142611

[10] Mackenbach JP (2019). Health inequalities: persistence and change in modern welfare states.

[11] Khang YH. The surprising result of manual workers in Korea enjoying lower mortality than non-manual workers is likely due to numerator-denominator bias. J Epidemiol Community Health. 2019; Available from: https://jech.bmj.com/content/early/2019/06/07/jech-2018-211715.responses?versioned=true#the-surprising-result-of-manual-workers-in-korea-enjoying-lower-mortality-than-non-manual-workers-is-likely-due-to-numerator-denominator-bias.

[12] Kim HR, Khang YH. Reliability of education and occupational class: a comparison of health survey and death certificate data. J Prev Med Public Health. 2005;38(4):443–8. 16358831

[13] Kweon S, Kim Y, Jang MJ, Kim Y, Kim K, Choi S (2014). Data resource profile: the Korea national health and nutrition examination survey (KNHANES). Int J Epidemiol.

[14] Korea Centers for Disease Control and Prevention (2008). Guideline for using raw data for national health and nutrition surveys IV (2007–2009).

[15] Shonkoff JP, Boyce WT, McEwen BS (2009). Neuroscience, molecular biology, and the childhood roots of health disparities. Jama..

[16] Bahk J, Yun SC, Kim YM, Khang YH (2015). Changes in the relationship between socioeconomic position and maternal depressive symptoms: results from the panel study on Korean children (PSKC). Matern Child Health J.

[17] Oakley L, Maconochie N, Doyle P, Dattani N, Moser K (2009). Multivariate analysis of infant death in England and Wales in 2005–06, with focus on socio-economic status and deprivation. Heal Stat Quarterly.

[18] Wickham S, Anwar E, Barr B, Law C, Taylor-Robinson D (2016). Poverty and child health in the UK: using evidence for action. Arch Dis Child.

[19] Currie C, Molcho M, Boyce W, Holstein B, Torsheim T, Richter M (2008). Researching health inequalities in adolescents: the development of the health behaviour in school-aged children (HBSC) family affluence scale. Soc Sci Med.

[20] Cavelaars AEJM, Kunst AE, Geurts JJM, Crialesi R, Grötvedt L, Helmert U (2000). Persistent variations in average height between countries and between socio-economic groups: an overview of 10 European countries. Ann Hum Biol.

[21] Li L, Manor O, Power C (2004). Are inequalities in height narrowing? Comparing effects of social class on height in two generations. Arch Dis Child.

[22] Ciol MA, Hoffman JM, Dudgeon BJ, Shumway-Cook A, Yorkston KM, Chan L (2006). Understanding the use of weights in the analysis of data from multistage surveys. Arch Phys Med Rehabil.

[23] Fujishiro K, Xu J, Gong F (2010). What does “occupation” represent as an indicator of socioeconomic status?: exploring occupational prestige and health. Soc Sci Med.

[24] Ministry of Employment and Labor (2015). Survey on labor conditions by employment type 2013.

[25] Lee HE, Kim H, Chung YK, Kang SK, Kim EA. Mortality rates by occupation in Korea: a nationwide, 13-year follow-up study. Occup Environ Med. 2016;73:329–35. 10.1136/oemed-2015-103192 PMC4853594 26920855

[26] Morassaei S, Smith PM (2011). Examining the relationship between psychosocial working conditions, physical work demands, and leisure time physical activity in Canada. J Occup Environ Med.

[27] Bonauto DK, Lu D, Fan ZJ (2014). Obesity prevalence by occupation in Washington state, behavioral risk factor surveillance system. Prev Chronic Dis.

[28] Schneider B, Grebner K, Schnabel A, Hampel H, Georgi K, Seidler A (2011). Impact of employment status and work-related factors on risk of completed suicide: a case–control psychological autopsy study. Psychiatry Res.

[29] Kim MH, Jung-Choi K, Jun HJ, Kawachi I (2010). Socioeconomic inequalities in suicidal ideation, parasuicides, and completed suicides in South Korea. Soc Sci Med.

[30] Bahk J, Lynch JW, Khang YH (2017). Forty years of economic growth and plummeting mortality: the mortality experience of the poorly educated in South Korea. J Epidemiol Community Health.

[31] Marmot MG, Bosma H, Hemingway H, Brunner E, Stansfeld S (1997). Contribution of job control and other risk factors to social variations in coronary heart disease incidence. Lancet..

[32] Bruner EJ, Kivimäki M, Siegrist J, Theorell T, Luukkonen R, Riihimäki H (2004). Is the effect of work stress on cardiovascular mortality confounded by socioeconomic factors in the Valmet study?. J Epidemiol Community Health.

[33] Kivimäki M, Leino-Arjas P, Luukkonen R, Riihimäi H, Vahtera J, Kirjonen J (2002). Work stress and risk of cardiovascular mortality: prospective cohort study of industrial employees. BMJ..

