# Supplementary material for: Analysis of factors contributing to occupational health inequality in Korea: a cross-sectional study using nationally representative survey data
Source: Arch Public Health. 2021 Jun 23;79:113. doi: 10.1186/s13690-021-00638-9 (PMC8220699; doi:10.1186/s13690-021-00638-9)
Supplement: Supplementary file 1 — Additional file 1: Table S1. Percentages and means of occupational class, childhood and adulthood socioeconomic position (SEP) indicators, and pathway variables for men aged 35–64: the 2007–2009 Korea National Health and Nutrition Survey (n = 4176). Table S2. Age-standardized percentages of childhood and adulthood socioeconomic position (SEP) indicators and pathway variables by occupational class for men aged 35–64: The 2013–2015 Korea National Health and Nutrition Survey (n = 4011). Table S3. Age-adjusted meansa of childhood and adulthood socioeconomic position (SEP) indicators and pathway variables by occupational class for men aged 35–64: the 2013–2015 Korea National Health and Nutrition Survey (n = 4011). [file 13690_2021_638_MOESM1_ESM.docx]

**ADDITIONAL FILE 1**

**Table S1. Percentages and means of occupational class, childhood and adulthood socioeconomic position (SEP) indicators, and pathway variables for men aged 35-64: the 2007-2009 Korea National Health and Nutrition Survey (n=4,176)**

| **Contributing factors** | | **%** | **Mean** |
| --- | --- | --- | --- |
| **Occupational**  **class** | Upper non-manual | 20.3 | - |
|  | Lower non-manual | 26.4 | - |
|  | Manual | 34.9 | - |
|  | Others | 18.5 | - |
| **Childhood**  **SEP** | **Parents’ education level** |  |  |
|  | Father's education (elementary school or less) | 52.6 | - |
|  | Mother's education (elementary school or less) | 67.7 | - |
|  | **Parents’ economic activity** |  |  |
|  | Absence of parents | 12.2 | - |
|  | Height (cm) | - | 169.5 |
| **Adulthood**  **SEP** | **Education level** |  |  |
|  | Elementary school or less | 13.1 | - |
|  | Middle school | 14.0 | - |
|  | High school | 37.7 | - |
|  | College or above | 35.2 | - |
|  | **Income** |  |  |
|  | Monthly household income (10,000 won) **^a^** | - | 315.1 |
| **Biological health**  **risk factors** | Body mass index (BMI) (≥30 kg/m^2^) | 3.4 | - |
|  | BMI (≤18) | 3.4 | - |
|  | BMI (kg/m^2^) | - | 24.3 |
|  | Blood pressure (≥140 mmHg) | 8.6 | - |
|  | Blood pressure (mmHg) | - | 118.5 |
|  | Serum total cholesterol (≥240 mg/dL) | 8.5 | - |
|  | Serum total cholesterol (mg/dL) | - | 191.6 |
|  | Serum glucose level (≥126 mg/dL) | 8.4 | - |
|  | Serum glucose level (mg/dL) | - | 101.0 |
| **Health**  **behaviors** | Current smoking | 46.5 | - |
|  | Smoking quantity per day (cigarette) | - | 18.5 |
|  | High-risk alcohol consumption **^b^** | 12.1 | - |
|  | Moderate level of physical activity | 13.7 | - |
|  | Exercise for weight control | 41.5 | - |
| **Psychosocial**  **factors** | Feelings of depression (more than 2 weeks) | 10.6 | - |
|  | Stress awareness | 28.1 | - |
|  | Marital status (Yes) | 94.9 | - |
|  | Suicidal ideation | 11.0 | - |
| **Work  environment ^c^** | Cleanliness and comfort | 65.4 | 2.8 |
|  | Dangerous | 35.0 | 2.2 |
|  | Time pressure | 35.4 | 2.3 |
|  | Authority | 74.8 | 3.0 |
|  | Respected and trusted | 80.9 | 3.0 |
|  | Long hours in an uncomfortable position | 22.9 | 2.1 |
|  | Carrying heavy objects | 25.6 | 2.0 |
|  | Hiding emotions | 33.0 | 2.3 |

a. Bottom-coding for less than 170,000 won per month and top-coding for more than 9 million won per month were applied.

b. Drinking more than seven drinks per day on average and almost every day in the past year

c. The proportion of those who responded “agree” or higher and the mean score of responses for each question about the working environment (strongly disagree, 1; disagree, 2; agree, 3; strongly agree, 4).

**Table S2. Age-standardized percentages of childhood and adulthood socioeconomic position (SEP) indicators and pathway variables by occupational class for men aged 35-64: The 2013-2015 Korea National Health and Nutrition Survey (n = 4,011)**

|  | | **Upper Non-manual** | | **Lower Non-manual** | | **Manual** | | **Others** | |
| --- | --- | --- | --- | --- | --- | --- | --- | --- | --- |
|  |  | **%** | **95% CI** | **%** | **95% CI** | **%** | **95% CI** | **%** | **95% CI** |
| **Childhood**  **SEP** | **Parents’ education level** |  |  |  |  |  |  |  |  |
|  | Father's education (elementary school or less ) | 37.4 | 33.5-41.3 | 44.8 | 41.3-48.4 | 55.9 | 52.8-59.1 | 50.6 | 44.4-56.8 |
|  | Mother's education (elementary school or less ) | 55.7 | 51.6-59.9 | 56.1 | 52.7-59.5 | 67.2 | 64.1-70.3 | 64.4 | 58.7-70.1 |
| **Adulthood**  **SEP** | **Education level** |  |  |  |  |  |  |  |  |
|  | Elementary school or less | 0.9 | 0.1-1.8 | 3.3 | 1.9-4.6 | 12.0 | 10.0-14.0 | 17.1 | 13.3-20.9 |
|  | Middle school | 1.1 | 0.3-1.8 | 5.8 | 4.0-7.5 | 16.1 | 14.0-18.2 | 11.8 | 8.7-14.8 |
|  | High school | 18.1 | 14.5-21.7 | 33.9 | 30.4-37.3 | 51.7 | 48.2-55.2 | 44.1 | 38.2-50.1 |
|  | College or above | 80.0 | 76.3-83.6 | 57.1 | 53.4-60.8 | 20.1 | 17.6-22.6 | 27.1 | 21.2-33.0 |
| **Biological health risk factors** | Body mass index (BMI) (≥30 kg/m^2^) | 4.5 | 2.9-6.1 | 4.7 | 3.2-6.2 | 4.8 | 3.4-6.2 | 6.1 | 3.4-8.9 |
|  | BMI (≤18 kg/m^2^) | 0.8 | 0.2-1.4 | 0.4 | 0.02-0.9 | 0.7 | 0.2-1.2 | 1.9 | 0.4-3.5 |
|  | Blood Pressure (≥140 mmHg) | 6.9 | 4.7-9.0 | 8.8 | 6.7-10.8 | 9.7 | 7.8-11.7 | 10.1 | 6.9-13.3 |
|  | Serum Total Cholesterol (≥240 mg/dL) | 9.4 | 7.0-11.7 | 9.1 | 7.1-11.1 | 9.4 | 7.5-11.4 | 12.2 | 8.3-16.1 |
|  | Serum Glucose Level (≥126 mg/dL) | 7.5 | 5.3-9.7 | 10.8 | 8.4-13.1 | 8.4 | 6.7-10.2 | 12.7 | 9.1-16.3 |
| **Health**  **behaviors** | Current smoking | 36.6 | 32.5-40.8 | 42.2 | 38.6-45.8 | 47.4 | 44.2-50.6 | 53.7 | 48.0-59.3 |
|  | High-risk alcohol consumption | 7.2 | 4.9-9.5 | 11.2 | 8.6-13.8 | 12.5 | 10.3-14.8 | 8.0 | 5.2-10.9 |
|  | Moderate level of physical activity (work) **^a^** | 7.4 | 5.2-9.7 | 7.1 | 5.2-8.9 | 11.5 | 8.9-14.0 | 9.0 | 4.8-13.2 |
|  | Moderate level of physical activity (leisure) **^a^** | 30.7 | 26.2-35.2 | 30.0 | 25.8-34.3 | 16.2 | 13.3-19.1 | 12.4 | 8.2-16.6 |
|  | Moderate level of physical activity (2013) | 2.3 | 1.0-3.6 | 3.6 | 2.1-5.0 | 1.7 | 1.0-2.5 | 3.3 | 1.2-5.4 |
|  | Exercise for weight control | 51.0 | 46.7-55.4 | 55.3 | 51.8-58.7 | 37.3 | 34.4-40.3 | 33.7 | 28.4-39.0 |
| **Psychosocial**  **Factors** | Feelings of depression (more than 2 weeks) | 2.5 | 1.3-3.8 | 3.1 | 2.0-4.2 | 6.1 | 4.6-7.6 | 9.2 | 6.2-12.2 |
|  | Stress awareness | 28.9 | 25.3-32.5 | 27.2 | 24.2-30.1 | 20.8 | 18.1-23.5 | 23.5 | 18.6-28.5 |
|  | Marital status (Yes) | 94.1 | 92.0-96.1 | 94.8 | 93.3-96.3 | 92.4 | 90.6-94.2 | 71.1 | 65.4-76.8 |
|  | Suicidal ideation | 1.3 | 0.5-2.3 | 0.9 | 0.2-1.6 | 3.2 | 2.0-4.5 | 4.7 | 2.9-6.5 |

a. Information on physical activity was obtained separately for work and leisure in 2014-2015.

**Table S3. Age-adjusted means^a^ of childhood and adulthood socioeconomic position (SEP) indicators and pathway variables by occupational class for men aged 35-64: the 2013-2015 Korea National Health and Nutrition Survey (n=4,011)**

|  | | **Upper Non-manual** | | **Lower Non-manual** | | **Manual** | | **Others** | |
| --- | --- | --- | --- | --- | --- | --- | --- | --- | --- |
|  |  | **Mean** | **95% CI** | **Mean** | **95% CI** | **Mean** | **95% CI** | **Mean** | **95% CI** |
| **Childhood SEP** | Height (cm) | 171.1 | 170.6-171.6 | 170.7 | 170.3-171.1 | 169.7 | 169.3-170.1 | 169.8 | 169.3-170.4 |
| **Adulthood**  **SEP** | Monthly household Income  (10,000 won) | 565.4 | 533.9-597.0 | 501.7 | 480.3-523.2 | 366.7 | 350.0-383.3 | 258.5 | 237.2-279.8 |
| **Biological health risk factors** | Body mass index (BMI) (kg/m^2^) | 24.5 | 24.3-24.7 | 24.7 | 24.5-24.9 | 24.5 | 24.3-24.7 | 24.4 | 24.1-24.7 |
|  | Blood pressure (mmHg) | 118.3 | 117.1-119.6 | 120.0 | 119.0-121.0 | 121.2 | 120.2-122.2 | 120.1 | 118.7-121.5 |
|  | Serum total cholesterol (mg/dL) | 194.3 | 191.2-197.4 | 193.1 | 190.5-195.6 | 193.2 | 191.1-195.3 | 192.6 | 189.1-196.1 |
|  | Serum glucose level (mg/dL) | 103.5 | 101.5-105.5 | 105.2 | 103.4-107.0 | 104.0 | 102.5-105.6 | 107.6 | 104.4-110.8 |
| **Health  behaviors** | Smoking quantity per day (cigarettes) | 5.2 | 4.4-6.0 | 6.1 | 5.4-6.7 | 8.3 | 7.7-9.0 | 8.6 | 7.7-9.5 |

a. Age-adjusted (50 years old) least squared means.
